# Roles of *Candida albicans* Gat2, a GATA-Type Zinc Finger Transcription Factor, in Biofilm Formation, Filamentous Growth and Virulence

**DOI:** 10.1371/journal.pone.0029707

**Published:** 2012-01-19

**Authors:** Han Du, Guobo Guan, Jing Xie, Yuan Sun, Yaojun Tong, Lixin Zhang, Guanghua Huang

**Affiliations:** 1 State Key Laboratory of Mycology, Institute of Microbiology, Chinese Academy of Sciences, Beijing, China; 2 Graduate University of Chinese Academy of Sciences, Beijing, China; 3 Chinese Academy of Sciences Key Laboratory of Pathogenic Microbiology and Immunology, Institute of Microbiology, Chinese Academy of Sciences, Beijing, China; Université de Nice-CNRS, France

## Abstract

*Candida albicans* is the most common human fungal pathogen, causing not only superficial infections, but also life-threatening systemic disease. *C. albicans* can grow in several morphological forms including unicellular yeast-form, elongated hyphae and pseudohyphae. In certain natural environments, *C. albicans* also exists as biofilms, which are structured and surface-attached microbial communities. Transcription factors play a critical role in morphogenesis and biofilm development. In this study, we identified four adhesion-promoting transcription factors (Tec1, Cph1, Ume6 and Gat2) by screening a transcription factor overexpression library. Sequence analysis indicates that Gat2 is a GATA-type zinc finger transcription factor. Here we showed that the *gat2/gat2* mutant failed to form biofilms on the plastic and silicone surfaces. Overexpression of *GAT2* gene promoted filamentous and invasive growth on agar containing Lee's medium, while deletion of this gene had an opposite effect. However, inactivation of Gat2 had no obvious effect on N-acetyl-glucosamine (GlcNAc) induced hyphal development. In a mouse model of systemic infection, the *gat2/gat2* mutant showed strongly attenuated virulence. Our results suggest that Gat2 plays a critical role in *C. albicans* biofilm formation, filamentous growth and virulence.

## Introduction

*Candida albicans* is the most common human fungal pathogen. With the increase in the number of immunocompromised patients, *Candida* infection is becoming more and more serious worldwide. *C. albicans* causes not only superficial infections, but also life-threatening systemic disease in immunocompromised hosts [1], [2]. Understanding the biology of this pathogen will definitely be helpful for developing new antifungal agents to combat this deadly pathogen.

*C. albicans* can grow in several morphological forms including unicellular yeast-form, elongated hyphae and pseudohyphae. The ability of switching between different growth modes is thought to be important for virulence [2], [3]. A variety of external and internal factors have been shown to regulate morphogenetic transition in this yeast. For example, the addition of inducers such as serum and N-acetylglucosamine (GlcNAc) and increases in temperature and pH can promote filamentous growth [4]. *C. albicans* morphogenesis is also regulated by a number of signal transduction pathways as well as a number of key regulators such as kinases and zinc finger transcription factors. Two major pathways, the cAMP/PKA mediated pathway and the Cst20-Ste11-Hst7-Cph1 pathway, have been intensively investigated [2], [5], [6], [7], [8].

*C. albicans* can also exist as biofilms, which are surface-associated microbial communities [9]. Many infections are related to the formation of biofilms on implanted medical devices [9], [10]. The morphological transition between cell types plays a critical role in *C. albicans* biofilm development under different circumstances. Baillie et al. has shown that the hyphal growth defective mutant only produces the basal layer, while the yeast growth defective mutant develops a thick, hyphal biofilm [11]. Deletion of *UME6*, encoding a filament-induced transcription factor, leads to hyphal growth and biofilm development defects [12]. Interestingly, the quorum sensing molecule farnesol, which is a filamentous growth inhibitor, also inhibits biofilm formation [13]. These facts indicate that the morphogenesis regulated pathways are also involved in regulation of biofilms in *C. albicans* [10].

The filamentous development regulator Tec1 is involved in biofilm formation by controlling the expression of Bcr1, a C2H2 zinc finger transcription factor [14].

Recently, Sahni et al. found that Tec1 specifically regulates the pheromone induced biofilm development of the *C. albicans* white phenotype by screening a library for 107 transcription factors [15]. In order to get more insights into the regulation of biofilm formation and morphorgenesis in *C. albicans*, we did a more extensive screen by using the same library with modified methods. In the Sanhi's study, they did the screen at 25°C and only cultured the cells for 16 hours [15]. The major modification of this screen was the increase of culture temperature to 30°C and extension of incubation time to 48 hours. In this study, we identified three more adhesion-promoting transcription factors (Cph1, Ume6 and Gat2) in addition to Tec1 which has also been discovered in the previous study [15]. Since the roles of Cph1, Ume6 and Tec1 in morphogenesis and biofilm formation have been intensively investigated, in this study we focused on the biological roles of Gat2, a GATA-type zinc finger transcription factor.

## Results

### Screen for the adhesion-promoting transcription factors

**Figure 1:**
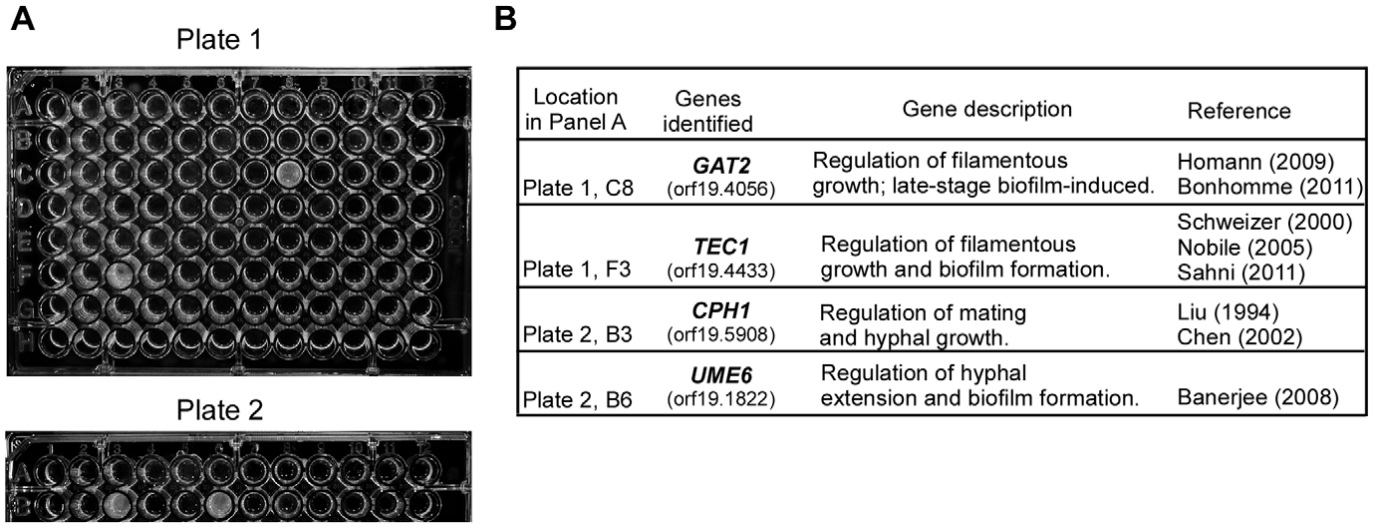
Biofilm development promoting transcription factors identified by a screen of an overexpression library. A. Screening of the library in 96-well plates. Overnight cultures were inoculated into a 96-well plate and then incubated for 48 hours with shaking at 30°C. The plates were washed with PBS and imaged. The parent strain transformed with the library was P37005, a natural *MTL* **a**/**a** isolate. B. Description of the four transcription factors identified.

*C. albicans* biofilm development includes a series of sequential steps: adherence→initiation→maturation→dispersal [10]. A lot of transcriptional regulators have been reported to control specific steps of the developmental process. By screening an overexpression library under the control of a doxycycline-inducible promoter [16], Sahni et al. have identified one adhesion-promoting transcription factor, that is, Tec1. Tec1 has been proved to be required for pheromone induced response in *C. albicans* white cells [15]. Given the complexity of biofilm development, we hypothesized that there would be more transcription factors or a transcription circuitry involved in the process. To prove this, we did another screen by using the same overexpression library constructed by the Soll lab [15]. We did the screen in 96-well plates at 30°C rather than at 25°C published in the previous study [15]. More importantly, we extended culture time to 48 hours since the culture time was critical for full biofilm development. These changes allowed mature biofilm development and lowered the threshold of screening the adhesion-promoting genes. Besides Tec1 identified in the early publication [15], we found 3 more adhesion-promoting transcription factors, including Cph1, Ume6 and Gat2 (**Figure 1**). Cph1 is a homolog of *S. cerevisiae* Ste12, which is required for mating and filamentous growth in the yeast. Deletion of *CPH1* results in hyphal growth defect on solid Spider medium [8] and blocks mating in *C. albicans* [17], but does not affect biofilm formation [5], [13], [15]. Ume6 has been shown to be required for hyphal extension, adherence to plastic and virulence [12]. Gat2 is a GATA-type zinc finger transcription factor, which has been shown to regulate filamentous growth on Spider medium in a high-throughput screen [18]. However, the molecular mechanism of filamentous growth regulation of Gat2 and its roles in invasive growth, biofilm formation and virulence remain unclear.

### Overexpression of adhesion-promoting genes (*GAT2*, *TEC1*, *CPH1* and *UME6*) induces filamentous growth

Filamentous growth ability directly relates to adhesion and biofilm formation in *C. albicans*. To further confirm the transcription factors we screened, we investigated the roles of the four transcription factors in promoting filamentous growth. Since the WT strain forms normal filamentous colonies at 37°C, the experiment was performed at 30°C, a temperature not favoring filamentous growth for *C. albicans*. As shown in **Figure S1**, overexpression of the transcription factors *GAT2*, *TEC1*, *CPH1 and UME6* in a WT strain promoted filamentous growth dramatically. The strain WT+ vector served as a negative control.

### Role of Gat2 in biofilm formation

**Figure 2:**
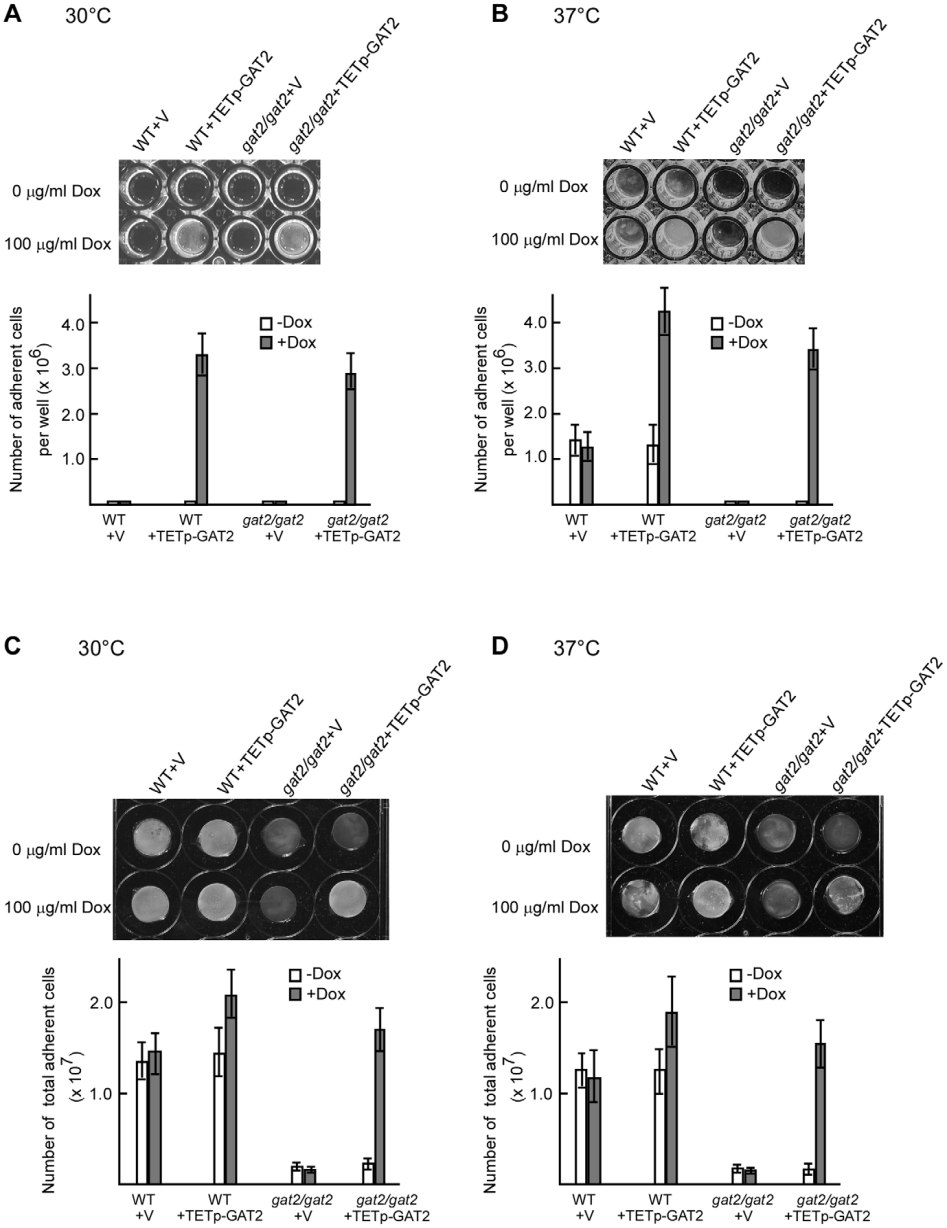
Role of Gat2 in biofilm formation. The parent strain for the *gat2/gat2* mutant and WT was SN152 (*MTL* **a**/α) [18]. A. Biofilm formation on the plastic bottoms of the strains (WT+ vector, WT+TETp-GAT2, *gat2/gat2* mutant+vector, *gat2/gat2* mutant+TETp-GAT2) at 30°C. Overnight cultures were inoculated into a 96-well plate and then incubated for 48 hours with shaking. The plates were washed with water and imaged. The numbers of cells adhered to the bottom represented three independent experiments. B. Biofilm formation on the plastic bottoms at 37°C. The same assay was used as in Panel A. C. Biofilm formation on silicone rubbers at 30°C. The silicone was incubated in a 24-well plate. The number of cells adhered to the silicone represented three independent experiments. D. Biofilm formation on silicone rubbers at 37°C. The number of cells adhered to the silicone represented three independent experiments.

To validate the adhesion-promoting activity of Gat2, we first tested the ability of adherence to the plastic 96-well plate bottoms in the *GAT2*-overexpression strain (WT+TETp-GAT2), *gat2/gat2* mutant (*gat2/gat2*+v) and the GAT2-reconstituted strain (*gat2/gat2*+TETp-GAT2). The wild type strain (WT+ v) carrying an empty vector served as control. At 30°C, all the strains were unable to adhere to the plastic bottom in the absence of doxycyline, while WT+TETp-GAT2 and *gat2/gat2*+TETp-GAT2 showed enhanced adhesion in the presence of 100 µg/ml doxycycline. The WT+ v and *gat2/gat2*+v strains failed to adhere to the plastic bottom even in the presence of 100 µg/ml doxycycline. The cells adhered to the bottoms were released and quantified by counting (**Figure 2A**).

At 37°C, in contrast to the WT+ v and WT+TETp-GAT2 strains, the *gat2/gat2*+v mutant was unable to form biofilms on the plastic bottom either in the presence or in the absence of 100 µg/ml doxycycline. However, the reconstituted strain *gat2/gat2*+TETp-GAT2 adhered to the bottom almost as strongly as the WT+TETp-GAT2 strain did in the presence of 100 µg/ml doxycycline (**Figure 2B**).

**Figure 3:**
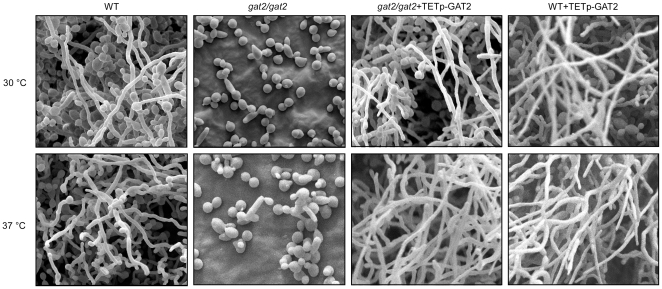
SEM of biofilms formed on silicone surfaces by the WT, *gat2/gat2* mutant, *gat2/gat2*+TETp-GAT2 and WT+TETp-GAT2 at 30 and 37°C. The images of *gat2/gat2*+TETp-GAT2 and WT+TETp-GAT2 under the inducing condition (100 µg/ml doxycycline) were shown. The data represented three independent experiments.

We also tested for the ability of biofilm development on a silicone cob in the strains as indicated in Figure 2C and D. The *gat2/gat2*+v mutant failed to form biofilm on the silicone surface at both 30°C and 37°C either in the presence or in the absence of doxycycline. The *gat2/gat2*+TETp-GAT2 strain formed normal biofilm as the reference strain did in the medium containing 100 µg/ml doxycycline at both temperatures (**Figure 2C, D**). At 30°C, the WT+ v formed normal biofilms on the silicone surface, although the ratio of hyphal cells to yeast cells was much lower than that at 37°C (data not shown). The cells adhered to the surface were quantified (**Figure 2C, D**). Visualization of scanning electron microscopy (SEM) confirmed the inability of biofilm development of the *gat2/gat2* mutant on silicone material surface (**Figure 3**). The *gat2/gat2*+TETp-GAT2 and WT+ TETp-GAT2 strains underwent robust filamentous growth and formed thick biofilms under inducing condition (**Figure 3**), while their phenotypes were similar to the *gat2/gat2* mutant and WT strains, respectively, under non-inducing condition (data not shown). The biofilm ultrastructure indicated that the biofilm formed by the wild type reference strain was a mixture of yeast cells and filamentous cells both at 30°C and at 37°C. However, the percentage of filamentous cells was much higher at 37°C than that at 30°C. The *gat2/gat2* mutant failed to undergo filamentous growth at 30°C and formed a few elongated cells at 37°C. Notably, the SEM images showed that the *gat2/gat2* mutant remained the basal level ability of adhering to the surface, although the number of adhered cells was much less than that of the WT.

### Deletion of *gat2/gat2* results in filamentous growth defect on Lee's medium plates

**Figure 4:**
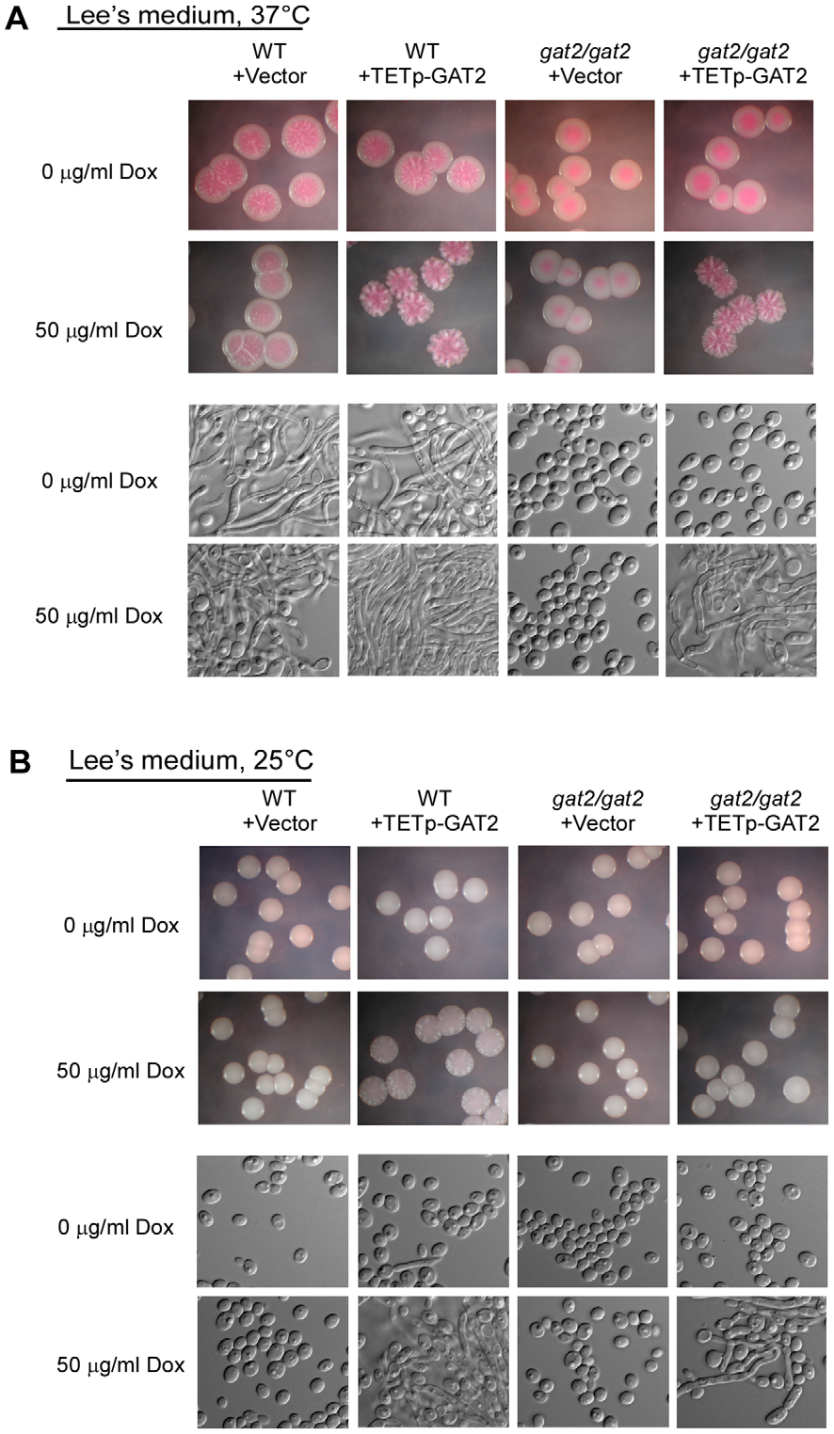
Gat2 is required for filamentous growth on Lee's medium. The strains indicated were incubated under non-inducing (0 µg/ml doxycycline) or inducing (50 µg/ml doxycycline) conditions. Colony and cellular pictures were shown. A. Filamentous growth at 37°C. B. Filamentous growth at 25°C.

Given the importance of hyphal development in biofilm formation, we hypothesized that Gat2 could play critical roles in filamentous growth. To test this, we first examined the hyphal growth ability of the strains WT+ v, *gat2/gat2*+v, WT+TETp-GAT2 and *gat2/gat2*+TETp-GAT2 on agar containing Lee's medium. In contrast to WT+ v, the mutant *gat2/gat2*+v was unable to form filamentous colonies at 37°C (**Figure 4A**). In the presence of 50 µg/ml doxycycline, the overexpression strain WT+TETp-GAT2 showed slightly stronger ability of filamentation than the WT+ v and *gat2/gat2*+TETp-GAT2 strains did (**Figure 4A**). At the cellular level, under inducing condition the WT+TETp-GAT2 was composed of over 98% of filamentous cells, while the WT+ v and *gat2/gat2*+TETp-GAT2 were composed of ∼80% and 95% of filamentous cells, respectively. We also did similar experiments at 25°C, a temperature unfavorable for *C. albicans* filamentous growth. We observed that only the overexpression strain was able to form star-like filamentous colonies under inducing condition at this temperature (**Figure 4B**). The *gat2/gat2*+TETp-GAT2 strain showed weak filamentous growth. The cellular images were also presented in **Figure 4B**. The additive effect of the endogenous and the ectopic expression of *GAT2* gene could result from the increased gene copies.

### Gat2 is not required for serum and GlcNAc induced filamentous growth

**Figure 5:**
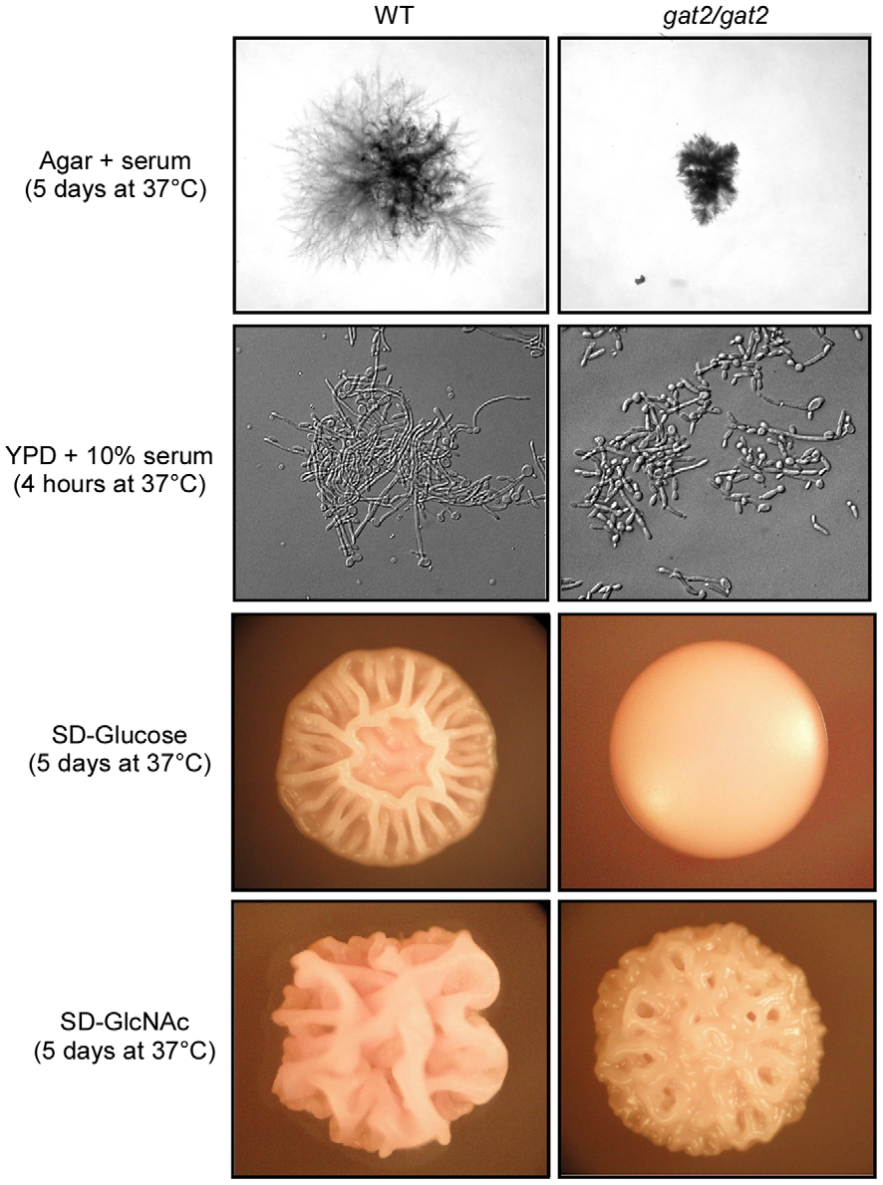
Deletion of *GAT2* impaired, but did not block filamentous growth induced by serum or GlcNAc. On solid agar, the strains were cultured for 5 days at 37°C and then imaged. In liquid YPD+serum medium, the strains were cultured for 4 hours at 37°C with shaking.

Different environmental cues induce filamentous growth through distinct pathways. Serum is thought to be the most potent hyphal inducer. We therefore tested whether Gat2 was also required for serum induced hyphal development. As shown in **Figure 5**, deletion of *GAT2* obviously attenuated filamentous growth ability but did not block the effect of serum induction both on agar and in liquid medium at 37°C. Compared to the reference strain, the *gat2/gat2* mutant formed small and less branched hyphal colonies on agar+serum plates. Consistently, the *gat2/gat2* mutant formed shorter hyphal cells in liquid YPD+10% serum medium.

GlcNAc is a powerful filamentous growth inducer in *C. albicans* [19]. Recently, we have reported that GlcNAc also regulates white-to-opaque transition via Ras1-cAMP/PKA pathway in this organism [20]. To investigate whether Gat2 was essential for GlcNAc induced yeast-to-hyphal transition, we incubated the reference and the mutant strains on SD-GlcNAc plates at 37°C. We found that the *gat2/gat2* null mutant formed obviously wrinkled hyphal colonies, although they were not highly wrinkled as those formed by the reference strain (**Figure 5**). These results indicate that Gat2 is not required for serum or GlcNAc induced filamentous growth.

### Gat2 is required for invasive growth

**Figure 6:**
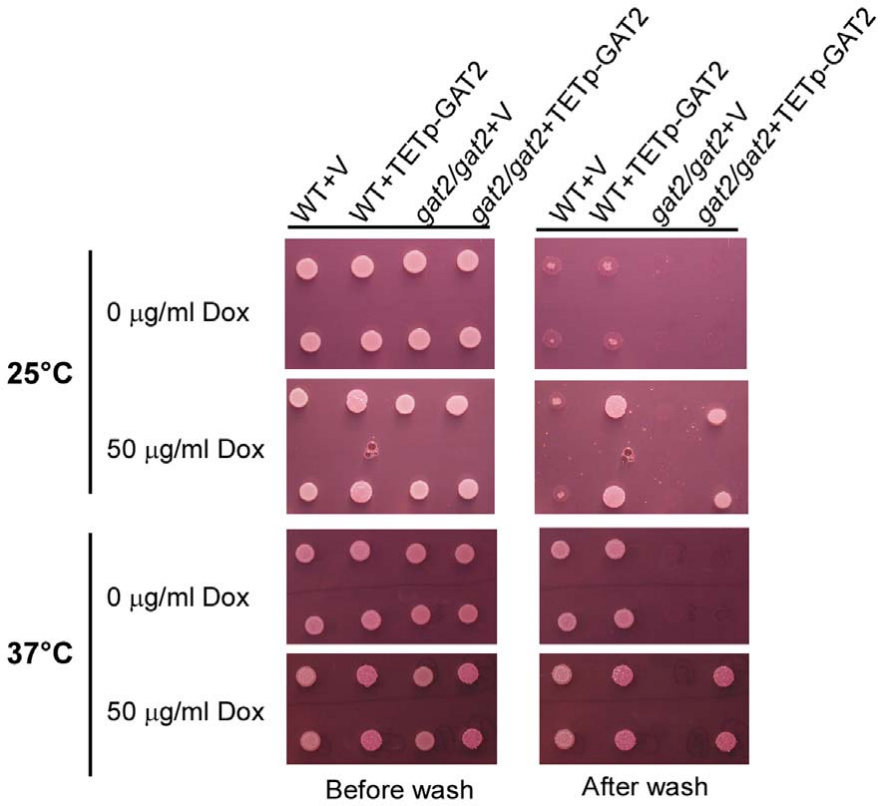
Role of Gat2 in invasive growth. The strains indicated were incubated under non-inducing (0 µg/ml doxycycline) or inducing (50 µg/ml doxycycline) conditions at 25°C or 37°C. The plates were imaged before and after washing.

The ability of *C. albicans* to undergo invasive growth is tightly linked to infection. To test the role of Gat2 in invasive growth, we performed the experiments at both 25 and 37°C. At 25°C, all the strains indicated in the figure were unable to undergo invasive growth under non-inducing condition, while the WT+TETp-GAT2 and *gat2/gat2*+TETp-GAT2 strains showed invasive growth under inducing condition (**Figure 6**). And the WT+TETp-GAT2 showed stronger invasive growth ability than the *gat2/gat2*+TETp-GAT2 strain did. At 37°C, under non-inducing condition the *gat2/gat2*+v and *gat2/gat2*+TETp-GAT2 failed to undergo invasive growth as the WT+ v and WT+TETp-GAT2 strains did. Under inducing condition, in contrast to the *gat2/gat2*+v strain, the *gat2/gat2*+TETp-GAT2 also underwent invasive growth as the WT+ v and WT+TETp-GAT2 strains did (**Figure 6**).

### Deletion of *GAT2* attenuates *C. albicans* virulence in a mouse model of systemic infection

**Figure 7:**
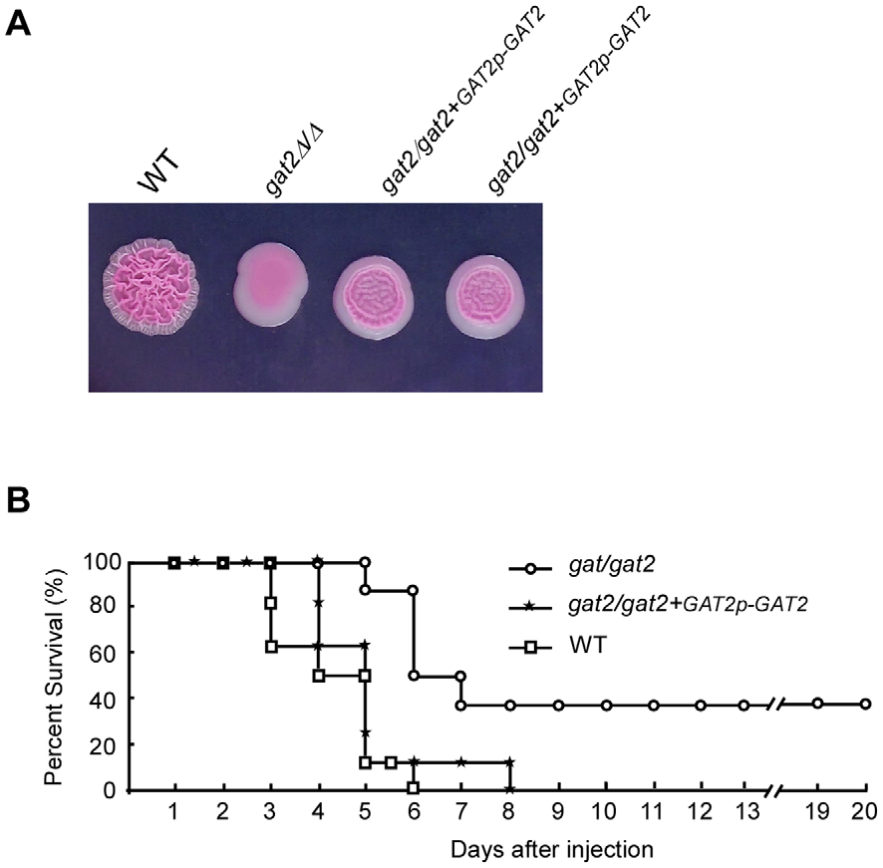
The *gat2/gat2* mutant is attenuated for virulence in a mouse systemic infection model. A. Filamentous growth of the reference strain, *gat2/gat2* mutant and the complemented strain *gat2/gat2*+GAT2p-GAT2. ∼3000 cells of each strain in 3 µL of ddH_2_O were dropped onto solid Lee's medium plates and incubated at 37°C for 3 days. Two independently isolates of the complemented strains were shown. B. Survival curves for the reference strain, *gat2/gat2* mutant and the complemented strain *gat2/gat2*+GAT2p-GAT2. For each strain, 8 mice were used for infection.

Filamentous morphogenesis is important for *C. albicans* virulence. Therefore, we tested the virulence of *gat2/gat2* mutant in a mouse model of systemic candidiasis. All of the mice injected with the WT reference strain died within 6 days, whereas the mice injected with *gat2/gat2* mutant died after a longer time period. Three mice (37.5%) were still alive at 20 days post-infection (**Figure 7**). To confirm the decrease of virulence was due to the deletion of *GAT2*, we constructed a complemented strain by inserting a fragment containing *GAT2* ORF and ∼400 bp promoter on the original *GAT2* locus. The filamentous growth ability of the complemented strains was restored (**Figure 7A**), although it was weaker than the WT. Notably, all the mice injected with the complemented strain were died within 8 days, suggesting that it was almost as virulent as the reference strain (**Figure 7B**). These results indicate that *gat2/gat2* mutant plays a role in virulence at least in a systemic infection model of mice.

## Discussion

Recently, Sahni et al. have identified *C. albicans* Tec1 as a key regulator of pheromone induced biofilm development through screening a transcription factor overexpression library [15]. To get more extensive insights into the molecular mechanism of biofilm and hyphal development, we did another screen using the same library with modified protocols in this study. We identified three more adhesion-promoting transcription factors (Cph1, Ume6 and Gat2) besides Tec1 that was also described in the previous report [15]. The roles of Tec1, Cph1 and Ume6 have been intensively investigated by us and others [8], [12], [15], [21]. Tec1 plays a critical role in both biofilm formation and filamentous growth [14], [15], [21]. The transcription of *TEC1* gene is regulated by Cph2, a Myc-bHLH family transcriptional activator of filamentous growth [22], while Tec1 controls the expression of biofilm regulator Bcr1 [14]. Cph1 regulates mating and filamentous growth in *C. albicans* [8], [17]. Ume6 has been proved to be required for hyphal extension, adhesion and virulence [12]. The GATA-type transcription factor Gat2 has been reported to be required for filamentous growth on Spider medium, a nutrient-poor medium for morphological analysis [18]. Consistent with previous study, we found that all the four transcription factors (Tec1, Cph1, Ume6 and Gat2) promote filamentous growth on solid Lee's medium. Additional experiments indicate that Gat2 plays important roles in biofilm formation, filmentous and invasive growth, and also virulence.

Biofilm development is controlled by a number of transcription factors including Tec1, Bcr1, Ume6, Efg1 and Zap1 [12], [14], [15], [23], [24]. Here, we added Gat2 to the list of biofilm regulators. Overexpression of *GAT2* in a wild type strain promotes adhesion and biofilm formation, while deletion of *GAT2* results in biofilm development defect.

Filamentous growth ability is thought to be important for biofilm development. Hyphae provide the structure integrity and multilayered architecture feature of muture biofilms [9]. Although Gat2 is not essential for serum- and GlcNAc-induced hyphal growth, deletion of *GAT2* notably attenuated the ability of hyphal growth stimulated by these two inducers, especially by serum. Remarkably, deletion of *GAT2* completely blocked filamentous growth in Lee's medium. These data suggest that different environmental cues activate filamentous growth via different pathways. Gat2 plays critical roles in Lee's medium induced morphogenesis and is at least partially involved in regulation of serum- and GlcNAc-induced yeast-to-hyphal transition. Consistently, we found that Gat2 is also not required for GlcNAc induced white-to-opaque switching in an *MTL*a/*a* strain (data not shown). Given the importance of filamentous growth ability in biofilm development, Gat2 possibly regulates biofilm development through filamentous growth control. Gat2 could be involved in regulation of the biofilm “initiation” and “maturation” steps, in which filamentous cells play critical roles [10].

In *S. cerevisiae*, ScTec1 binds to the promoter of *ScGAT2* [25]. By sequence analysis, we found two putative Tec1 binding sites on the promoter region of *GAT2* gene ( TCATTCT and ACATTCT ) [26]. Interestingly, *tec1/tec1* mutant showed similar phenotypes on SD-glucose and SD-GlcNAc media as *gat2/gat2* mutant did. Both Tec1 and Gat2 were not required for filamentous growth induced by GlcNAc, but were essential for full hyphal development on SD-glucose medium (data not shown). Similar roles of Gat2 and Tec1 in adhesion and GlcNAc induced filamentous growth suggest that Gat2 possibly functions downstream of Tec1 in regulation of morphogenesis and biofilm development.

Our findings reveal that Gat2 is involved in regulation of biofilm development, morphogenesis and virulence. On Lee's medium plates which are characterized by neutral pH and poor nutrient, deletion of *GAT2* gene completely blocked filamentous and invasive growth. However, Gat2 is not essential for filamentous growth induced by some environmental cues, such as serum and GlcNAc. We propose that Gat2 specifically regulates morphogenesis in some host niches.

## Materials and Methods

### Strains and growth conditions

The transcription factor overexpression library was constructed by the Soll lab [15]. The *gat2/gat2* mutant and the reference strain were requested from the Johnson's lab [18]. While the strains used in **Figure 1** and **Supplemental figure S1** were homozygous at *MTL* locus (**a**/**a**), all the others were *MTL* heterozygous (**a**/α). Solid YPD medium (20 g/L Difco peptone, 10 g/L Yeast extract, 20 g/L glucose, 20 g/L Agar) and Lee's medium supplemented with 5 µg/ml phloxin B were used for routine growth. Lee's medium, SD-glucose, SD-GlcNAc and agar+serum plates were used for filamentous development [27]. In the SD-GlcNAc medium, 2% GlcNAc replaced glucose as carbon source. K_2_HPO_4_ (2.5 g/L) was added to the SD-glucose and SD-GlcNAc media for pH maintenance. The pH of Lee's and SD media was adjusted to 6.8 with 10% HCl.

To construct the complemented strain *gat2/gat2*+GAT2p-GAT2, we first generated a plasmid pGAT2res for transformation of the *gat2/gat2* mutant. A fragment of the 3-UTR of *GAT2* and a fragment containing the *GAT2* ORF and ∼400 bp of the promoter region were subsequently inserted into the plasmid pNIM1, and yielded pGAT2res. The complemented strain was generated by transforming the *gat2/gat2* mutant with *Sal*I digested pGAT2res fragments. The oligonucleotides used for PCR were listed below:

GAT2-3F-xho: 5-aatcaaCTCGAGcgctgtaaattatatcctga-3;

GAT2-3R-Bglsal: 5-aatcaaAGATCTatGTCGACatatctcagtgcagaaacagg-3;

GAT2-5F-Sal: 5-aatcaaGTCGACaacccgtttaacatttgcagc-3;

GAT2-5R-Bgl: 5-aatcaaAGATCTaattagatgtgtacattaatttctatg-3.

### Biofilm assay

Biofilm experiments were performed as described previously with slight modifications [15]. For adhesion to the plastic bottoms, cells were cultured in Costar 96-well Cell Culture Plates at temperatures indicated in the main text. After 48 hours of incubation with shaking, the wells were gently washed with 1× PBS (phosphate-buffered saline). The bottoms were imaged. Biofilm growth on silicone material was performed as reported with slight modification [14]. Briefly, cells were incubated in a well of a 24-well cell culture plate containing a round silicone block with a diameter of 1 cm. Silicone blocks were cut from Cardiovascular Instrument silicone sheets. After 48 hours of culture, the silicone blocks were carefully washed and taken out for imaging. After gently washing, the cells adhered to the bottoms of 96-well plates or silicone material were treated with trypsin and collected for quantitation.

### Scanning electron microscopy (SEM)

For SEM, we developed *C. albicans* biofilms on silicone blocks. The SEM assay was performed as previously reported [11]. Briefly, the samples were gently washed with 1× PBS and fixed with 2.5% glutaraldehyde. Then, the samples were washed three times with 0.1 M Na_3_PO_4_ buffer (pH 7.2), dehydrated inincreasing concentrations of ethanol (30% - 50% - 70% - 85% - 95% - 100%) and coated with gold. The surface of the biofilm was imaged with a scanning electron microscopy (FEI QUANTA 200).

### Invasive and filamentous growth assays

Lee's medium plates with or without doxycycline as indicated were used for invasive growth. 3 µl of liquid medium containing 2×10^4^ cells was dropped onto the agar for 2 days (at 37°C) or 5 days (at 25°C) of incubation. The plates were imaged before and after washing with H_2_O. Agar containing serum, Lee's medium, SD-glucose or SD-GlcNAc medium was used for filamentous growth analysis. The colonies were imaged after 5 days' culture at temperatures indicated.

### Virulence experiments

The virulence of *C. albicans* strains was performed as reported by Chen *et al.* [28]. ICR male mice (18–22 g) were used for the systemic infection experiments. 100 µl of 1× PBS containing 2×10^6^ cells was injected into each mouse. All animal experiments were performed according to the guidelines approved by the Animal Care and Use Committee of the Institute of Microbiology, Chinese Academy of Sciences (permit number: IMCAS2011002). The present study was approved by the Committee.

## Supporting Information

Figure S1 **Ectopic expression of adhesion-promoting genes (** ***GAT2*** **,** ***TEC1*** **,** ***CPH1*** **and** ***UME6*** **) induces filamentous growth in** ***C. albicans*** **.** The strains were cultured at 30°C for 5 days and imaged.
(TIF)
