# Supplementary figures and images for: Roles of Candida albicans Gat2, a GATA-Type Zinc Finger Transcription Factor, in Biofilm Formation, Filamentous Growth and Virulence
Source: PLoS One. 2012 Jan 19;7(1):e29707. doi: 10.1371/journal.pone.0029707 (PMC3261855; doi:10.1371/journal.pone.0029707)

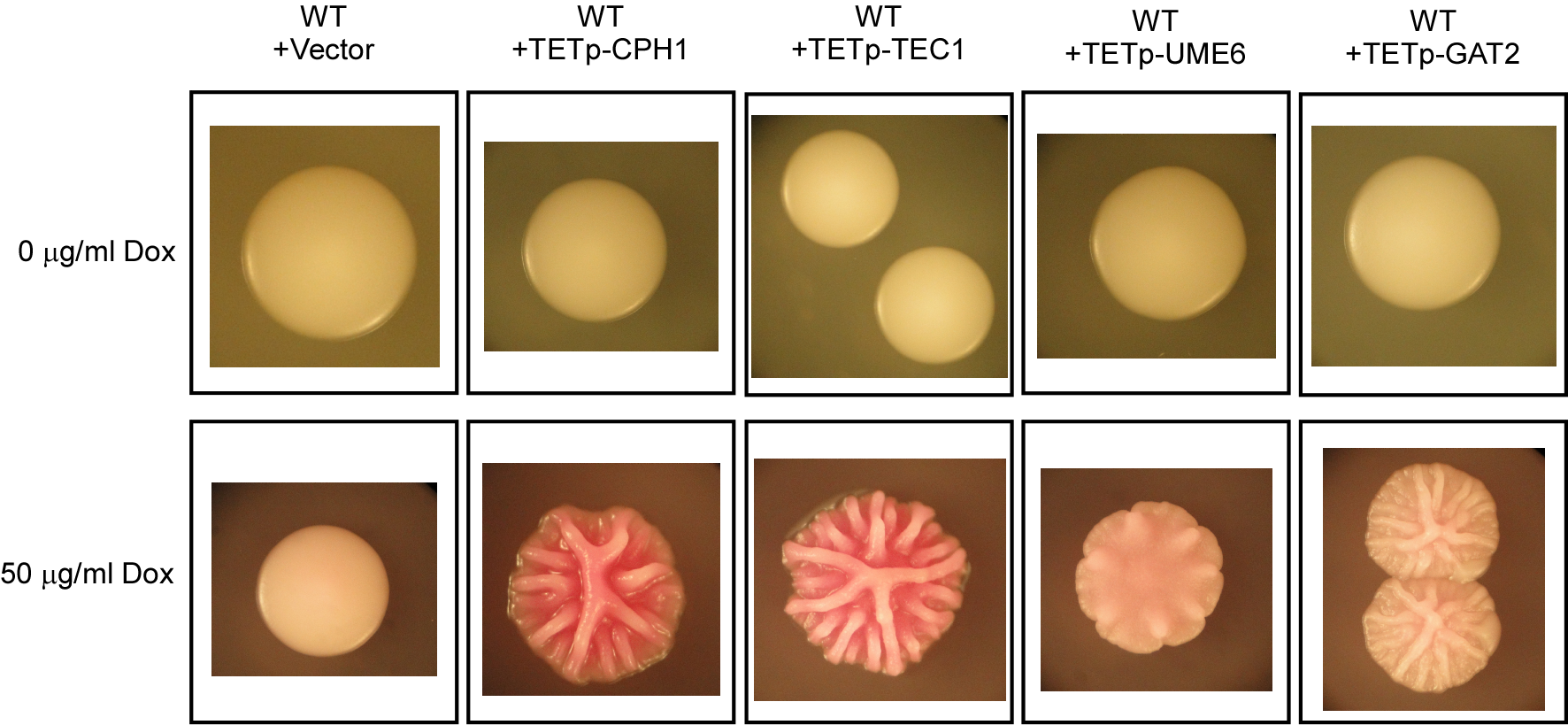

Supplement: Figure S1 — Ectopic expression of adhesion-promoting genes ( GAT2 , TEC1 , CPH1 and UME6 ) induces filamentous growth in C. albicans . The strains were cultured at 30°C for 5 days and imaged. (TIF) [file pone.0029707.s001.tif]
